# Cortical Inhibition and Plasticity in Major Depressive Disorder

**DOI:** 10.3389/fpsyt.2022.777422

**Published:** 2022-01-26

**Authors:** Jesminne Castricum, Tom K. Birkenhager, Steven A. Kushner, Ype Elgersma, Joke H. M. Tulen

**Affiliations:** ^1^Department of Clinical Genetics, Erasmus University Medical Center, Rotterdam, Netherlands; ^2^Department of Neuroscience, Erasmus University Medical Center, Rotterdam, Netherlands; ^3^Department of Psychiatry, Erasmus University Medical Center, Rotterdam, Netherlands; ^4^ENCORE Expertise Center for Neurodevelopmental Disorders, Erasmus Medical Center, Rotterdam, Netherlands

**Keywords:** transcranial magnetic stimulation, cortical plasticity, motor evoked potential (MEP), cortical inhibition, major depressive disorder (MDD)

## Abstract

**Background:**

Major depressive disorder (MDD) is a severe psychiatric disorder that is associated with various cognitive impairments, including learning and memory deficits. As synaptic plasticity is considered an important mechanism underlying learning and memory, deficits in cortical plasticity might play a role in the pathophysiology of patients with MDD. We used Transcranial Magnetic Stimulation (TMS) to assess inhibitory neurotransmission and cortical plasticity in the motor cortex of MDD patients and controls.

**Methods:**

We measured the cortical silent period (CSP) and short interval cortical inhibition (SICI), as well as intermittent theta-burst stimulation (iTBS), in 9 drug-free MDD inpatients and 18 controls.

**Results:**

The overall response to the CSP, SICI, and iTBS paradigms was not significantly different between the patient and control groups. iTBS induction resulted in significant potentiation after 20 mins in the control group (*t*_(17)_ = −2.8, *p* = 0.01), whereas no potentiation was observed in patients.

**Conclusions:**

Potentiation of MEP amplitudes was not observed within the MDD group. No evidence was found for medium-to-large effect size differences in CSP and SICI measures in severely depressed drug-free patients, suggesting that reduced cortical inhibition is unlikely to be a robust correlate of the pathophysiological mechanism in MDD. However, these findings should be interpreted with caution due to the high inter-subject variability and the small sample size.

**Significance:**

These findings advance our understanding of neurophysiological functioning in drug-free severely depressed inpatients.

## Introduction

Major depressive disorder (MDD) is a severe psychiatric disorder with a prevalence of 4.7% worldwide (1). MDD comprises a depressed mood and loss of interest or pleasure in life activities. The majority of MDD patients also suffer from cognitive dysfunction (2, 3). Previous research showed various cognitive impairments in MDD patients, including deficits in memory, attention, language, and visual-motor speed (4, 5).

The mechanism underlying the cognitive deficits associated with MDD remains poorly understood. The cellular mechanism of learning and memory is believed to depend on the ability to induce long-lasting changes in synaptic efficacy. The ability of synapses to enhance their strength or efficacy of synaptic transmission over time, i.e., long-term potentiation (LTP), has been well studied in animals. Although findings of cortical plasticity in humans show important parallels with LTP, there is a lack of evidence that the cortical potentiation is due to synaptic changes. Therefore, in the literature, the term LTP-like plasticity is often used when referring to lasting cortical plasticity. A previous study reported significant performance impairment in three learning tasks in MDD patients (5). Based on neurophysiological findings, previous studies hypothesized that cortical plasticity is impaired in patients with MDD (6–9). These studies made use of Transcranial Magnetic Stimulation (TMS), a neurophysiologic technique to assess inhibitory and excitatory neurotransmission in the motor cortex via single-pulse stimulations, as well as the modulation of cortical excitability via TMS paradigms (10). Reduced cortical plasticity was shown in 23 and 27 MDD patients, taking psychotropic drugs at the time of measurement, in response to the paradigm of paired associative stimulation (PAS) (6, 7). Recently, one study investigated cortical plasticity in 11 drug-free MDD patients with the intermittent theta-burst stimulation (iTBS) paradigm (8). The iTBS paradigm bears a strong resemblance with the methodology used in *ex vivo* preclinical studies to measure LTP (11). In addition, due to its shorter duration and low stimulus intensity, the iTBS paradigm is less demanding than PAS and therefore more suitable to use in severely depressed patients. Vignaud et al. (8) showed impaired cortical plasticity upon iTBS in MDD treatment-resistant patients, although they observed high variability in the response to iTBS. There are only a few studies of cortical plasticity in MDD patients, in which the effect of psychoactive drug use and the variation in depression severity has remained unclear.

Cortical plasticity in patients with MDD seems to be modulated by gamma-aminobutyric acid (GABA), the main inhibitory neurotransmitter in the central nervous system (12). GABAergic interneurons inhibit other neurons in the cortex to coordinate cortical activity and modulate synaptic plasticity. Several preclinical studies have shown that GABAergic deficits play a role in cognitive dysfunction associated with MDD traits such as anxiety and distortion of attention to threat cues (13, 14). In addition, Stockmeier et al. (15) observed a reduction in GABAergic connections postmortem in the hippocampus of 19 MDD patients. Following the theory that synaptic plasticity is essential for learning and memory, cognitive dysfunction in MDD could be caused by deficits in the GABAergic neurotransmitter system.

GABA deficits in MDD have been extensively studied in preclinical and treatment studies, including TMS (12). The TMS paradigms short-interval intracortical inhibition (SICI) and cortical silent period (CSP) are sensitive to changes in GABA-mediated inhibition. Previous studies have shown that the response to the SICI or CSP paradigms can be increased by GABA_A_ or GABA_B_ receptor agonists (16, 17). However, TMS studies of GABA-mediated cortical inhibition in MDD patients have yielded contradictory findings. The response to the SICI was significantly increased, and CSP was significantly shortened, in 20 drug-free patients with treatment-resistant MDD, indicating reduced cortical inhibition in MDD (18). Conversely, the response to the SICI was not significantly different in 16 MDD patients (19). Moreover, 16 depressed patients had significantly prolonged CSP, suggesting an increase of cortical inhibition in MDD (20). However, most of these patients either received psychoactive drugs at the time of the study (19, 20) or had a non-response to treatment with antidepressants (18). Notably, drugs that act on the central nervous system can strongly influence the response to TMS paradigms (21).

Considering the prevalence of cognitive dysfunction clinically reported in MDD patients, and the inconsistent TMS findings in MDD patients, further clarification of the presence of underlying neurophysiological deficits in drug-free severely depressed patients is relevant. Interestingly, it has been shown that age similarly affects GABAergic cortical inhibition as late-life depression (22). Therefore, it is highly important to age-match patients and controls. Additionally, the effect of confounders as experimental factors, sleepiness, time of day, and gender should be considered due to high variability in TMS measures observed in healthy individuals (9, 23). Hence, we examined both cortical inhibition and cortical plasticity in drug-free severely depressed inpatients compared to age-matched neurotypical controls using the TMS paradigms SICI, CSP, and iTBS, in which potential confounders were systematically considered.

## Methods

### Subjects

In this study, we included drug-free MDD patients and controls matched for age and gender. Participants were included with an age between 18 and 85 years. Patients were included if they had a confirmed diagnosis of major depression according to the criteria of the DSM V (24) and were being free of psychoactive drugs (see Table 1 for “days without medication”). Patients were excluded if they had other somatic or psychiatric comorbidities as bipolar disorder or psychotic symptoms. Additionally, patients had no neurological diseases as Parkinson's disease or Alzheimer's disease, or any brain pathology as a cerebrovascular accident. Lastly, patients with an indication for acute electroconvulsive therapy were excluded. Controls were included if they had a score on the Beck Depression Inventory (BDI) (26, 27) below 9 and were medication free (excluding contraceptives). Controls had no current or history of medical, psychiatric, or neurological disorders. Furthermore, subjects had no neurological illness that could affect the motor system and used no psychoactive drugs. Subjects met the criteria for undergoing a TMS measurement (28, 29). Inpatients with MDD were recruited by a senior psychiatrist from the depression unit of the Department of Psychiatry at the Erasmus University Medical Center. Recruitment of unaffected controls took place through online advertisements.

**Table 1:** Characteristics of MDD patients.

**MDD patients**	**Age**	**Gender**	Edu^*^	**HAM-D**	**Psychoactive medication <1 month**	**Dose in mg (times per day)**	**Days without medication**	**Mean half-life (hrs)**
01	58	F	-	17	NA	NA	NA	
02	59	M	-	17	Clomipramine	75 (2) 25 (1)	20 16	21
03	46	F	6	20	Olanzapine Lamotrigine	5 (1) 50 (2)	11 4	30 33
04	44	F	-	24	Lorazepam	1 (2)	2	12–16
05	70	F	2	20	Venlafaxine	375(1) 75 (1) 37.5(1)	13 6 1	5
06	47	M	7	16	NA	NA	NA	
07	47	M	3	18	Lorazepam Venlafaxine	0.5 (1) 37.5 (1)	2 2	12–16 5
08	56	F	3	14	Pregabaline Lithium Nortriptyline	75 (1) 200 (1) 600 (1) 25 (1)	0 13 11 13	6 12–48 26
09	66	F	3	29	Propranolol Haloperidol Trazodon Temazepam Lorazepam Lithium Escitalopram	10 (2) 0.5 (1) 50 (1) 20 (1) 10 (1) 1 (1) 400 (1) 200 (1) 10 (1)	1 7 9 13 10 10 13 10 13	3–6 12–38 8 7–11 12–16 12–48 30

*Age, gender, education, Hamilton score (HAM-D), and medication specifications per patient with MDD*.

* *Education, level of education using the International Standard Classification of Education (ISCED) (25). “-”, level of education unknown*.

*MDD, major depressive disorder; Edu, education level; HAM-D, Hamilton score; F, female; M, male; NA, not applicable*.

We achieved our *a priori* sample size estimations (30) based on data from previous studies. To detect a medium to large-sized effect for cortical plasticity (η^2^ = 0.12) with a power of 80% and a significance level of 0.025 (Bonferroni corrected), we needed a sample size of minimal 7 subjects per group (patient and control groups) (7, 8). To detect a large-sized effect for cortical inhibition (SICI: η^2^ = 0.22; CSP: d = 1.02) with a power of 80% and a significance level of 0.025, we needed a sample size of minimal 7 and 17 subjects per group, respectively (18).

This study was conducted following the Declaration of Helsinki (2013) and was approved by the Dutch Central Medical Ethics Committee of the Erasmus Medical Center Rotterdam.

### Procedures

Participants were screened before the start of the TMS measurements using the questionnaires Transcranial magnetic stimulation Adult Safety Screen (TASS) (29), Beck Depression Inventory (BDI) (controls) (26), and Hamilton Rating Scale for Depression (HAM-D) (patients) (31) (see Section Questionnaires). We classified the level of education using the International Standard Classification of Education (25). We started the TMS measurements at noon for all subjects after they had a light lunch. Subjects had their eyes open and arms at rest while sitting in a comfortable chair. We recorded motor evoked potentials (MEPs) from the left first dorsal interosseous (FDI) muscle using electromyography (EMG) with silver/silver chloride electrodes in belly-tendon recording technique. We used a universal amplifier (ANT Neuro, Enschede, The Netherlands). Data was filtered online with a 20–2,000 Hz band-pass filter and a 50 Hz notch filter, and raw data was stored for offline analysis. TMS stimulations were given by a TMS stimulator (MagPro X100 with MagOption; MagVenture, Denmark) via an eight-shaped stimulation coil (MC-B70, MagVenture, Denmark) placed on the scalp. The handle of the coil was held in a posterolateral direction at an angle of 45° from the midline. First, we determined the optimal positioning of the coil on the primary motor cortex in the right hemisphere (i.e., the hotspot) in accordance with the reference point of the FDI. The reference point was defined on the right hemisphere as the place at 10% of the ear-to-ear span lateral to Cz. We placed randomly around this reference point TMS stimulations to define the hotspot with the highest peak-to-peak amplitude of the MEP in the FDI muscle. Throughout the experiment, the coil was held at the hotspot using a 3D neuronavigation (Visor2XT). The resting motor threshold (RMT) was defined with a maximum likelihood threshold-hunting procedure (32). RMT is the stimulus intensity that elicited MEPs of > 50 μV with a 50% probability. The RMT measurement was repeated at 3-time points to control for changes over time. Sleepiness was also measured at these time points with the Karolinska sleepiness scale (KSS), a self-report questionnaire on a nine-point Likert scale (33). Furthermore, throughout the experiment, single-pulse stimulations were given with a stimulus intensity that elicited a mean and median between 800 and 1,200 μV ± SD <1/2 of the mean (SI1mV). The SI1mV was determined by the mean of 10 stimulations with increasing stimulus intensity starting from the RMT (34, 35). The differences in MEP amplitude as a response to the TMS paradigms SICI, CSP and iTBS were studied.

#### Questionnaires

The TASS is a validated questionnaire to screen TMS candidates consisting of 15 questions (29). Positive answers to one or more questions do not represent absolute contraindications to TMS. The BDI is a 21-question multiple-choice self-report inventory to measure the severity of depression in controls (26). The HAM-D is a 17-item questionnaire (31), commonly used to rate the severity of depression. Right-handedness was determined for each person with the Edinburgh Handedness Inventory (EHI) (36).

#### TMS Measurements

##### SICI

Short interval cortical inhibition (SICI) is a paired-pulse TMS paradigm that measures cortical inhibition. In this paradigm, a subthreshold pulse of 80% of RMT is followed by a pulse at SI1mV after an interstimulus interval of <6 ms. The SICI has been reliable and reproducible within individuals (37). We performed in random order 17 paired stimulations with the conditioning pulse at 80% of RMT, and 13 single stimulations at the SI1mV. The SICI paradigm used an interstimulus interval of 3 ms. The difference in MEP amplitude between the response to paired and single pulses was used to estimate cortical inhibition.

##### CSP

During the cortical silent period (CSP) paradigm, the FDI was tonically contracted with 20% of maximum voluntary strength using a hand-held pinch gauge (B&L Engineering; Santa Ana, CA, USA). The CSP is determined from the time the single suprathreshold TMS pulse is given until EMG activity reappears after the MEP. We visually identified the reappearance of EMG activity. Single pulses consisted of 10 pulses at 120% of RMT with an inter-stimulus interval of 6 secs (38). The CSP has been shown to have good test-retest reliability (39).

##### ITBS

Theta burst stimulations are repetitive bursts of 3 stimuli at a frequency of 50 Hz repeated at 5 Hz. In the intermittent TBS (iTBS) paradigm, a train of TBS of 2 secs was repeated every 10 secs for a total of 190 secs (11). We used a stimulus intensity of 70% of RMT for the iTBS and recorded 20 single pulses at SI1mV before iTBS and at 0, 10, 20, 30 mins after iTBS modulation (11, 40–42). The original protocol described the use of a stimulus intensity of 80% active motor threshold (AMT). However, in accordance with some previous studies (40, 41), we used the intensity of 70% RMT to avoid muscle contraction prior to iTBS that could affect the TBS-aftereffects (43, 44). The burden of the use of 70% RMT is also lower than the use of higher intensities. Changes in mean MEP amplitude after iTBS induction compared to the mean MEP amplitude before iTBS induction are assumed to reflect changes in cortical plasticity.

### Data Analyses

EMG data was online continuously recorded with Visor software (Visor2XT). The raw data from the Visor program were analyzed using Matlab (Matlab, version 2019b). First, all the traces were detrended if a linear trend was present. Secondly, a bandpass filter between 20 and 2,000 Hz and a notch filter at 50 Hz with an elliptic design was applied to the raw EMG data. Thereafter, traces were discarded if the peak-to-peak amplitude of the EMG activity in rest was higher than 70 μV and a standard deviation higher than 25 μV within a 50 ms pre-trigger interval (45, 46). We used a range of 10 μV lower than the cut-off values to visually detect technical artifacts or excessive background EMG activity during rest. TMS responses of one-time point within a participant were discarded if more than 50% of the epochs were discarded at that time point (42). Lastly, MEP amplitude and peak latencies were calculated within a time window of 0.2–48 ms. We defined the MEP onset automatically and visually within 20–35 ms after the TMS trigger. If the data was not normally distributed, MEP amplitudes were transformed with a square root transformation to reduce right skewness (47, 48). Statistical analyses were performed using the (transformed) MEPs in IBM Statistics SPSS (version 25).

We tested for differences in age, gender, educational attainment, and sleepiness between groups with an independent *t*-test, a Chi-square test, and non-parametrically with a Mann-Whitney *U*-test, respectively. The change over time in RMT during the experiment was tested with a repeated-measures ANOVA. The difference in CSP durations between groups was evaluated with an independent *t*-test. We performed a mixed model ANOVA to compare mean MEP amplitudes between groups during the paradigms. For the SICI outcome, the main effects were condition and group, and we tested whether a change in MEP amplitude is caused by the interaction between condition (paired or single pulses; within-subjects factor), and group (MDD or control; between-subjects factor). For the iTBS outcome, the main effects were time and group, and we tested the interaction between time (T0, T1, T2, T3; within-subjects factor) and group (between-subjects factor) on MEP amplitude.” In addition, we tested separately the responders to iTBS in both groups, classified as a minimal increase of 10% in MEP amplitude after iTBS induction at T0, T1, T2, or T3 (40, 49). Relationships between confounding factors such as age and the HAM-D score and the main outcomes were evaluated using Pearson or Spearman's rho (r_s_) correlation coefficients, respectively, and *p*-values were corrected for multiple comparisons with the Bonferroni correction.

## Results

**Figure 1:**
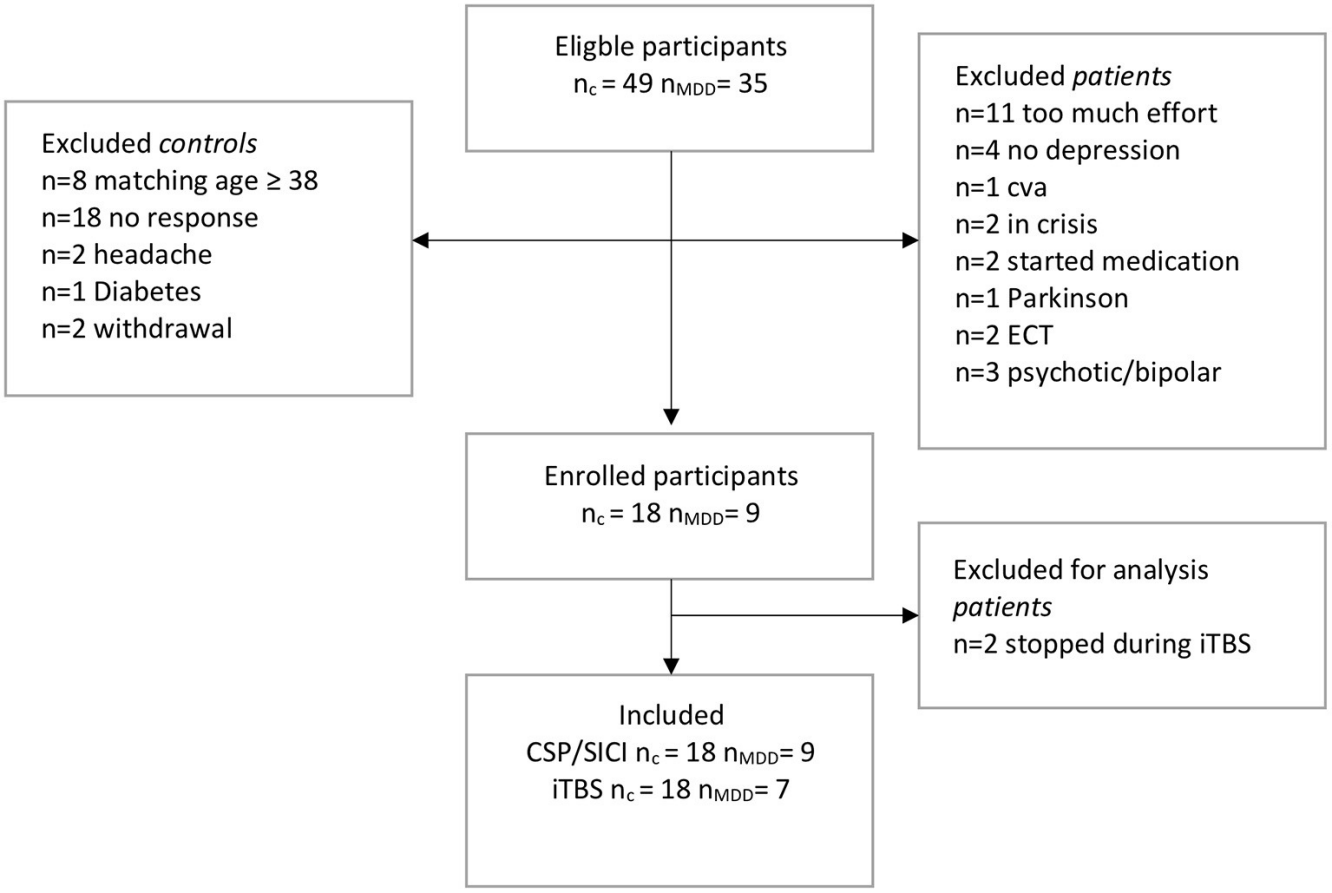
Flow-chart of inclusions. c, control; MDD, patients with major depressive disorder; TMS, transcranial magnetic stimulation; SICI, short interval cortical inhibition; iTBS, intermittent theta-burst stimulation; CSP, cortical silent period; CVA, cerebrovascular accident; ECT, electroconvulsive therapy.

In total, 35 eligible drug-free patients with MDD were invited of which 11 subjects declined participation, 13 subjects were excluded due to other (psychiatric) comorbidities, and 2 subjects had no diagnosis of severe depression (Figure 1). In total, we included 9 patients. In addition, 49 eligible control subjects were invited of which 18 subjects were included (n_c_ = 18, n_MDD_ = 9) (Figure 1). Two patients withdrew during the iTBS paradigm. They reported fatigue and requested to stop the experiment.

Age was not significantly different between the patient group (M = 54.8 ± 9.4) and the control group (M = 51.1 ± 10.6) (*t*_age__(25)_ = −0.9, *p* = 0.4). Gender was not significantly different between the groups ($\chi_{\text{gender}}^{2}$ = 1.2, *p* = 0.3). The level of education also was not significantly different between the groups (U = 37.0, *p* = 0.3). The mean HAM-D score for patients was 19.4 ± 1.5. The mean drug-free period was 10.5 ± 8.1 days at the time of testing. Before the drug-free period, patient psychotropic usage in the month prior included: tricyclic antidepressants (*n* = 2; clomipramine, nortriptyline), selective serotonin reuptake inhibitors (*n* = 3; trazodone, escitalopram), selective serotonin norepinephrine inhibitors (*n* = 1, venlafaxine), antipsychotics (*n* = 2; olanzapine, haloperidol), anti-epileptics (*n* = 2; lamotrigine, pregabalin), benzodiazepines (*n* = 4; lorazepam, temazepam), lithium (*n* = 2) and beta-blockers (*n* = 1; propranolol) (Table 1). The mean sleepiness score during the measurements was significantly higher (i.e., less alert) in the patient group than in the control group (U = 31.5, *p* = 0.02).

RMT was not different between the groups (*t*_RMT(25)_ = −0.8, *p* = 0.4) and did not change over time (*F*_(2, 38)_ = 0.12, *p* = 0.9). The mean amplitude of MEPs at SI1mV was similar for the patient group and the control group (M_c_ = 967 ± 297; M_MDD_ = 837 ± 340, *t*_(25)_ = 1.0, *p* = 0.3). Stimulus intensities were similar for both groups (*t*_SI1mV(25)_ = −0.4, *p* = 0.7) (Table 2).

**Table 2:** Demographics, educational attainment, and variables during transcranial magnetic stimulation (TMS) measurements (Mean ± SD) of the major depressive disorder (MDD) group and the control group separately.

	**MDD group**	**Control group**
	**(*n* = 9)**	**(*n* = 18)**
**Demographics**		
Age in years	54.8 ± 9.4	51.1 ± 10.6
Gender: Male in % (^#^)	33 (*3*)	56 (*9*)
**Educational attainment**		
Educational attainment (median, range)	3.0, 2–7	6.0, 2–7
**Sleepiness (Median, range)**		
Median KSS^*^	7.0, 1–9	3.0, 1–5
**During TMS measurements**		
RMT %MSO	50.4 ± 9.9	47.3 ± 9.4
SI_1mV_ %MSO	59.2 ± 13.6	57.4 ± 12.0
Mean amplitude of MEPs at SI_1mV_	837.5 ± 340.6	966.5 ± 297.8

# *, Number of subjects; KSS, Karolinska sleepiness scale; TMS, transcranial magnetic stimulation; RMT, Resting Motor Threshold; SI_1mV_, Stimulus Intensity at 1 mV; MSO, Maximum Stimulator Output; MEP; motor evoked potential; MDD, major depressive disorder*.

* *Significantly different between the patient and control group (p-value <0.05)*.

### Cortical Inhibition

**Figure 2:**
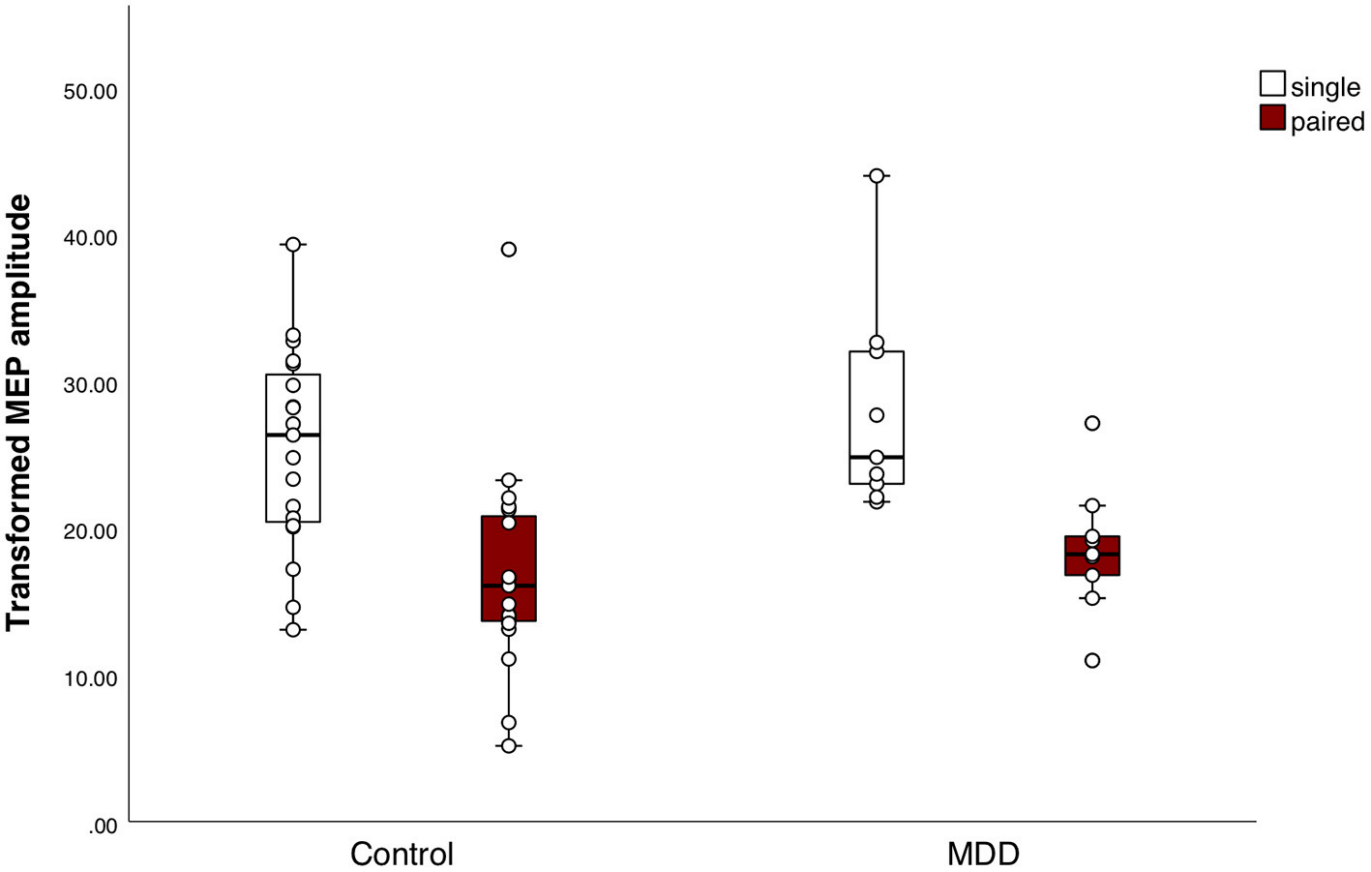
Response to the short interval cortical inhibition (SICI) paradigm. Boxplots of square-root (sqrt) transformed mean motor evoked potential (MEP) amplitudes per subject in response to the SICI, for both groups separately. Mean MEP amplitudes in response to the single pulses or paired pulses did not differ between the major depressive disorder (MDD) group and the control group. Mean MEP amplitude differed significantly between conditions, indicating that the SICI paradigm sufficiently inhibited the MEPs in both groups (*F*_(1, 25)_ = 44.0, *p* < 0.001, η^2^ = 0.6).

**Figure 3:**
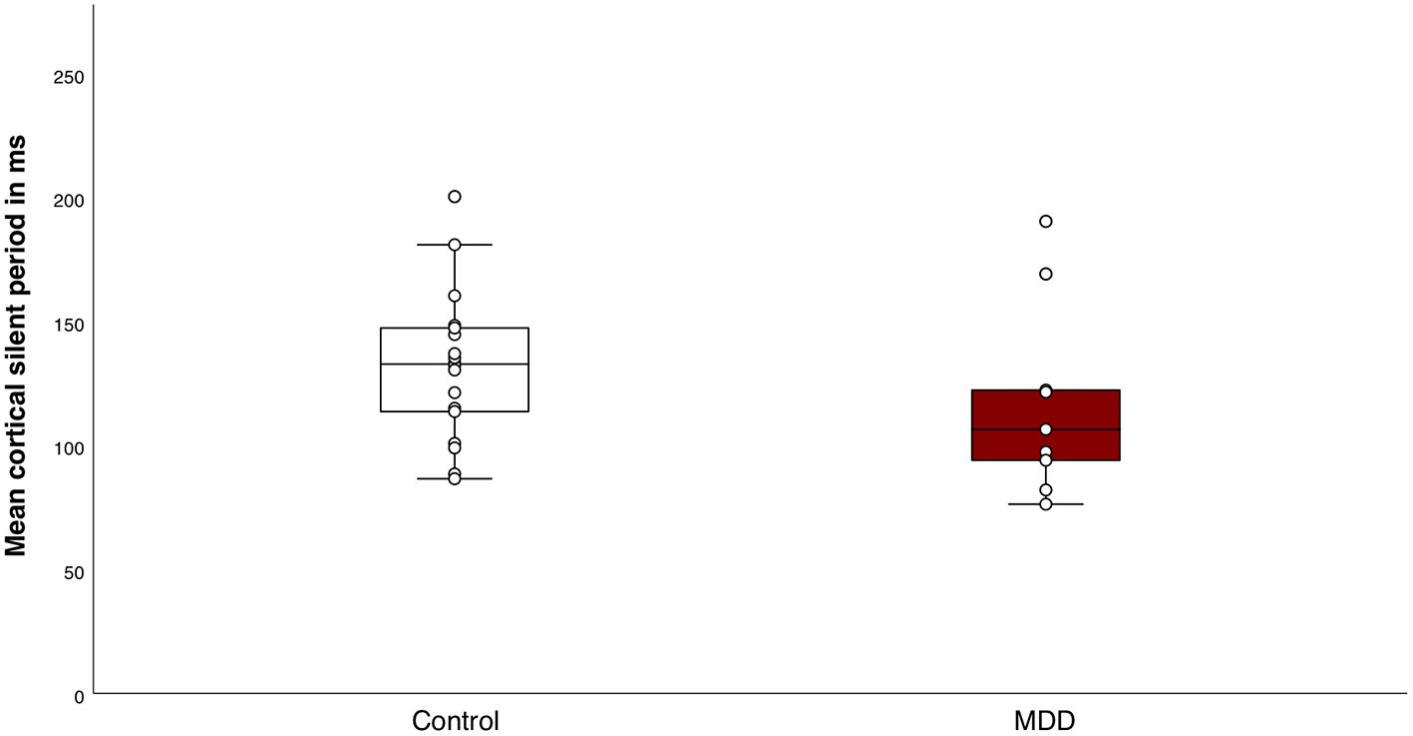
Response to the cortical silent period (CSP) paradigm. Boxplot of individual means of CSP duration for the control group and the major depressive disorder (MDD) group. There were no significant differences in mean CSP duration between the groups (*t*_(25)_ = 1.1, *p* = 0.3).

The mean MEP amplitude of singe pulse stimulations was not different between groups (*t*_(25)_ = −1.0, *p* = 0.4). Mean MEP amplitude differed significantly between conditions, indicating that the SICI paradigm sufficiently inhibited the MEPs in both groups (*F*_(1, 25)_ = 44.0, *p* < 0.001, η^2^ = 0.6) (Figure 2). We did not find a significant group effect (*F*_(1, 25)_ = 0.8, *p* = 0.4) or interaction effect between group and conditions (*F*_(1, 25)_ = 0.3, *p* = 0.6). The SICI paradigm inhibited the MEPs in both groups equally. Additionally, there was no significant difference in mean CSP duration between the MDD group and the control group (M_c_ = 132.0 ± 30.0; M_MDD_ = 117.7 ± 38.8) (*t*_(25)_ = 1.1, *p* = 0.3) (Figure 3).

### Cortical Plasticity

**Figure 4:**
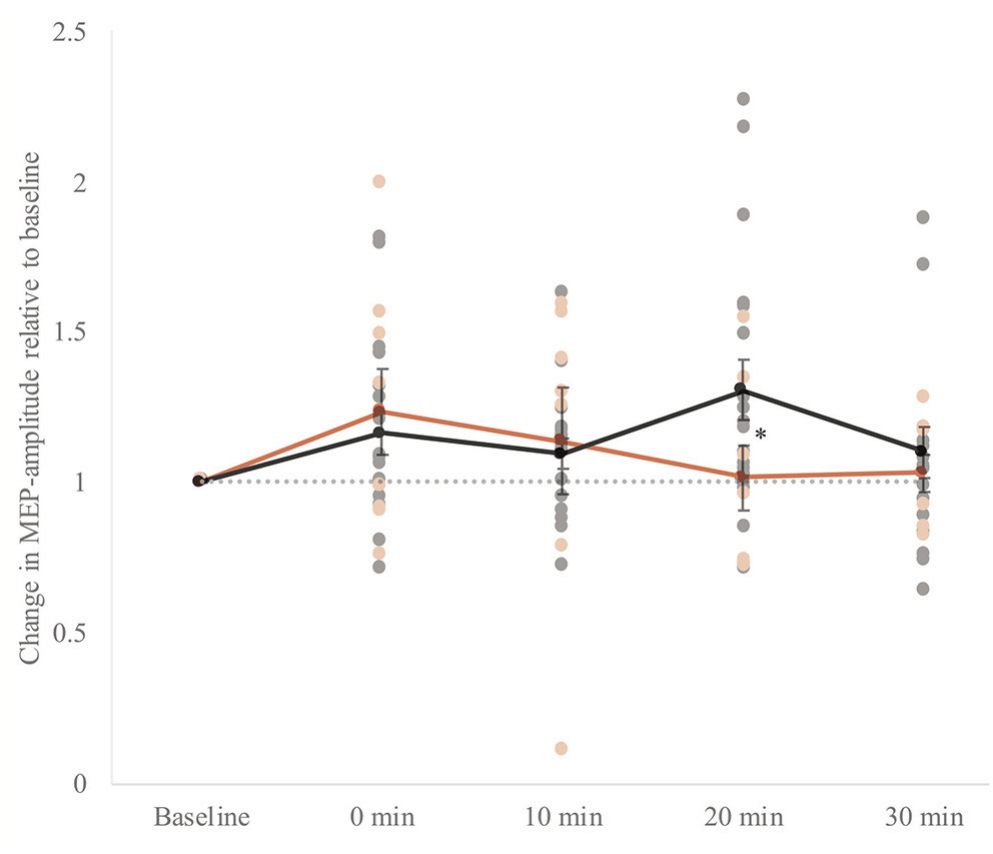
Whole group analysis of cortical plasticity. The change in MEP amplitude (motor evoked potential) amplitudes ±SEM of the response upon induction of intermittent theta-burst stimulation (iTBS). Baseline: mean MEP in response to single pulses directly before iTBS. 0–30 min: mean MEP in response to single pulses four times within 30 mins after stimulation: 0, 10, 20, and 30 mins after stimulation. MEPs measured after stimulation were significantly higher in both groups (*F*_time (4, 88)_ = 3.4, *p* = 0.01, η^2^ = 0.13). A gray dotted horizontal line at value 1.0 is plotted to interpret the potentiation. The individual data points are also presented. We did not find a significant group effect (*F*_group (1, 22)_ = 0.09, *p* = 0.8). However, there was an interaction effect between group and time (*F*_(4, 88)_ = 2.5, *p* = 0.05, η^2^ = 0.10). In the control group, within-group analyses by means of t-tests showed that the MEP amplitude following iTBS was significantly higher 20 mins after stimulation (*i.e*., T3) (*t*_(17)_ = −2.8, *p* = 0.01) indicated with an asterisk (*). In the patient group, within-group analyses showed that the MEP amplitude following iTBS was not significantly higher for any time point compared to baseline.

At baseline, MEPs in response to single-pulse TMS before iTBS induction were not different between the groups (*t*_(24)_ = −0.5, *p* = 0.6). Mixed model ANOVA revealed that MEPs measured after stimulation were significantly higher in both groups (*F*_time(4, 88)_ = 3.4, *p* = 0.01, η^2^ = 0.13 i.e., small effect). We did not find a significant group effect (*F*_group (1, 22)_ = 0.09, *p* = 0.8), and an interaction effect on trend level between group and time (*F*_(4, 88)_ = 2.5, *p* = 0.05). In the control group, within-group analyses by means of *t*-tests showed that the MEP amplitude following iTBS was significantly higher 20 mins after stimulation (i.e., T3) (*t*_(17)_ = −2.8, *p* = 0.01). In the patient group, within-group analyses showed that the MEP amplitude following iTBS was not significantly higher for any time point compared to baseline (Figure 4). The standard errors of the mean were large in both groups indicating a high inter-subject variability.

Additionally, the number of responders to iTBS, classified as a minimal increase of 10% in MEP amplitude after iTBS induction at T0, T1, T2 or T3, was not significantly different between groups (control = 83%; MDD = 67%, χ^2^(1) = 0.96, *p* = 0.3). Mixed model ANOVA of the responder group revealed similar results as the whole group analysis (*F*_time(4, 76)_ = 4.6, *p* = 0.002, η^2^ = 0.2 i.e., small effect; *F*_group (1, 19)_ = 0.4, *p* = 0.5; *F*_interaction (4, 76)_ = 2.4, *p* = 0.05). In the control group of responders, within-group analyses showed a significant potentiation of the MEP amplitude following iTBS at 0–20 mins after stimulation (*t*_T1(14)_ = −3.7, *p* = 0.002; *t*_T2(14)_ = −3.3, *p* = 0.005; *t*_T3(14)_ = −4.4, *p* = 0.001). In the patient group of responders, within-group analyses showed that the MEP amplitude following iTBS was not significantly higher for any time point compared to baseline.

### Correlations

There were no significant correlations between the severity of the depression of inpatients measured with HAM-D and the main outcomes nor between the potential confounders age, gender, educational attainment, or sleepiness and the main outcomes. There were no significant correlations between the HAM-D score of inpatients and the duration of the CSP (*r*_s_ = −0.2, *p* = 0.6), the MEPs inhibited by SICI (r_s_ = −0.7, *p* = 0.07), nor the increase of the MEPs induced by iTBS (r_s_T0_ = 0.8, *p* = 0.08). We also did not find significant correlations in either group between age and the duration of the CSP (*r* = −0.1, *p* = 0.5), the MEPs inhibited by SICI (*r* = 0.05, *p* = 0.8), or the increase of the MEPs induced by iTBS (r_T0_ = −0,08, *p* = 0.7). There were no significant correlations between sleepiness and the increase of the MEPs induced by iTBS (r_s_T0_ = −0.2, *p* = 0.4).

## Discussion

Whether changes in neurophysiological measures are present in drug-free severely depressed patients causing the cognitive deficits remains poorly understood. We examined TMS-based measures of cortical inhibition and plasticity in drug-free severely depressed inpatients and controls. Previous studies showed inhibitory GABAergic dysfunction in MDD patients, which might consequently affect cortical plasticity. Based on these previous findings, we expected alterations in the response to the inhibitory TMS measures CSP and SICI, as well as a reduced response to the plasticity measure iTBS in MDD patients. Potentiation of MEP amplitudes was not observed within the MDD group. No evidence was found for medium-to-large effect size differences in CSP and SICI measures in severely depressed drug-free patients, although a high inter-subject variability was noted in both groups.

We found no evidence for differences in CSP and SICI measures reflecting GABA-mediated inhibition in MDD patients. In both the MDD and control groups, the MEP amplitudes were similarly inhibited in response to the paradigms. Although previous studies were inconsistent, several studies showed a trend toward decreased inhibition in MDD patients as measured with the CSP and SICI (18, 19, 50). The CSP paradigm is considered to be a very robust paradigm used in the study of the pathophysiology of several psychiatric disorders such as obsessive-compulsive disorder and schizophrenia (51, 52). The CSP is associated with deficits in GABA_B_ receptor-mediated inhibitory neurotransmission (17, 53). It was shown in a meta-analysis that the CSP duration was shortened in MDD patients compared to controls (50). Contradictory, in the present study, we did not find a significant difference in CSP duration between the patient and control group, although our MDD group was quite small according to *a priori* sample size estimations. However, previous studies did not use a standardized protocol to measure the CSP, causing difficulties in comparing the findings. Previous studies used different stimulus intensities (range 110–200% of RMT), different strengths of muscle contraction and measured from different hemispheres (i.e., dominant vs. non-dominant). The increasing stimulus intensity is known to increase the CSP duration (54). Nevertheless, the stimulus intensity of 120% of RMT in the present study provides a reliable and informative CSP (55). In addition, this stimulus intensity was used in the present study, because MDD patients can be more sensitive to potential discomfort as induced by increasing stimulus intensities of the TMS. The strength of muscle concentration was relatively low to avoid fatigue of the muscle, although CSP duration seems not to be affected by the strength of muscle contraction (55). Lastly, we stimulated the non-dominant hemisphere due to less cortical inhibition in the dominant hemisphere than in the non-dominant hemisphere (56).

In the present study, the SICI paradigm sufficiently inhibited the MEPs in both groups, but the amount of inhibition was not different between the groups in contrast to previous studies. Although Levinson et al. (19) showed no difference in the SICI response between unmedicated MDD patients (i.e., without medication for at least 1 month) and controls, previous studies did find a difference between treatment-resistant MDD patients and controls (18, 19). Possibly, these patients had a more severe illness causing more inhibitory deficits, although the HAM-D score was not different between the unmedicated and treatment-resistant depressed patients. In the present study, all patients were inpatients admitted to the hospital for a longer period, nevertheless, some patients suffered from moderate depression (Table 1). It is important to note that the treatment-resistant patients measured in Levison et al. (19) used medication during the study which could have influenced the results (21), whereas in our study, the patients were drug-free. The SICI and CSP might not only measure inhibitory processes mediated by GABA, but also measure processes that interact with the GABAergic neurotransmitter system or processes from independent inhibitory pathways in the primary motor cortex (19). Despite evidence of reduced GABA levels in depressed patients by studying the treatment of selective serotonin reuptake inhibitor and electroconvulsive therapy (ECT) (57, 58), the present findings are in line with some previous studies that looked into GABA-related deficits (59, 60). More specifically, Knudsen et al. (60) found no differences in GABA levels between depressed and healthy participants before or after ECT treatment. Bhagwagar et al. (61) suggested that reduced GABA levels might be associated with a trait of vulnerability to mood disorder based on findings in recovered patients, instead of a direct neurochemical correlate of MDD.

The second main finding of our study is the absence of potentiation of the MEP amplitudes upon iTBS induction within the MDD group. We found that the MEP amplitude had a marked effect in potentiation following iTBS induction at 20 mins in the control group, as the MEP amplitude decreased quickly after induction to baseline values in the MDD group. This effect was even stronger when only including the data of the responders: we observed a significant increase in MEP amplitude immediately after iTBS-induction up to 20 mins in the control group, but there was no potentiation in the patient group. It is important to note that the percentage of responders was not significantly different between the groups, nevertheless, the group sizes were quite small. To our knowledge, only one study investigated cortical plasticity in 11 drug-free treatment-resistant depressive patients with the iTBS paradigm (8). Interestingly, our findings are consistent with Vignaud et al. (8) who showed a significant potentiation following iTBS induction at 20 mins in controls and no potentiation in MDD patients. However, in the present study, we did not find a significant overall group effect, and our findings should be investigated further to confirm plasticity deficits in MDD patients. Furthermore, reduced cortical plasticity was shown in MDD patients taking psychoactive drugs at time of measurement, in response to the paradigm paired associative stimulation (PAS) (6, 7). The present study showed comparable TMS results in drug-free MDD patients using a more favorable method, iTBS, due to its lower stimulation intensity and duration. The effect of iTBS induction seems to depend on N-methyl-D-aspartate (NMDA) receptors (62), of which alterations in the levels have been shown in the brain of depressive patients (63).

Our findings should be interpreted with caution due to the high inter-subject variability. To reduce the variability, we increased the single pulse stimulations per timepoint (8, 42). Moreover, we standardized experimental settings such as time of day, and we matched for sex and age (64). It has been shown that age similarly affects GABAergic cortical inhibition as late-life depression (22). Additionally, sleepiness was measured to consider its potential confounding on the outcome. Patients were less alert than the control group, although this could be associated with their MDD symptoms. Nevertheless, there were no significant correlations between the main outcomes and the sleepiness score. We also used the personalized SI_1mV_ to avoid ceiling and floor effects within subjects instead of a standard percentage of the RMT. This could be optimized further, however, by using an input-output curve of the individual stimulus intensity (65). Nevertheless, the high inter-individual variability in TMS responses could be the result of genetics, the current state of neuronal activity, or the recruitment of early or late indirect waves (I-waves) (66–68). Future research should address the high inter-subject variability in the response to iTBS to clarify cortical plasticity in drug-free MDD patients.

The key strength of the present study is the relative homogeneity of the patient group. All patients had severe symptoms and were inpatients at the time of the study, admitted to the hospital for extended stays for treatment of a major depressive disorder. Patients had no psychiatric comorbidities or discernible brain pathology. Additionally, patients were free of psychoactive medication for an average of 10 days prior to the study. Although the severity of the depression in patients was slightly lower (M = 19.4 ± 1.5) than previous studies that found large effects on inhibitory measures (M = 21.2 ± 6.0; 21.1 ± 1.1) (18, 69), the scores were still within the range of moderate to severe depression. Nevertheless, Lewis et al. (70) showed that the severity of the depressive symptoms might correlate with the degree of neurophysiological dysfunction in a pediatric sample. In the present study, we did not find such a correlation, in line with studies of an adult sample with MDD (19, 20).

Our study is limited by its small sample size, the location of stimulation on the human cortex, the variability in the duration of the drug-free period, and the variability in the use of psychoactive drugs before the drug-free period. The number of inpatients was low due to the known inherent lack of motivation among patients with severe MDD, the low number of admissions to the hospital, and the short amount of time to test the patients between admission and the start of treatment with antidepressants. We achieved our *a priori* sample size estimations (30) for the iTBS and SICI outcomes based on data from previous studies (7, 18, 19), but we were unable to reproduce the large differences in TMS measures reported in previous studies. Nevertheless, a larger sample size might reduce the observed high inter-subject variability (71), although the effect of age on the outcome measures should be still taken into account. Furthermore, our measures are limited to the primary motor cortex, while we are interested in neurophysiological processes that are not involved in motor function. The dorsolateral prefrontal cortex might be more interesting to stimulate with TMS combined with electroencephalography to study the pathophysiology of major depressive disorder (72). Potentially, the findings of neurophysiological processes in the primary motor cortex could be translated to other cortices. Lastly, the duration of the drug-free period was rather short in some of the patients. Medication could affect the outcome measures in patients that had a short wash-out period of the psychoactive drugs. We carefully acknowledged medication half-life in the study design. Furthermore, the patients that had a short wash-out period of the medication before the measurement used medication with no known effect on the inhibitory TMS measures (21). Only one patient with a short wash-out period used a benzodiazepine agonist that increases GABA-mediated inhibition, but no clear deviations were found in this patient in relation to the other study subjects.

Future research should investigate further whether deficits in cortical inhibition is a robust pathophysiological mechanism in MDD, and if the absence of within-subject potentiation is still present with a larger sample size in drug-free patients. Perhaps, in the future, treatment could be optimized by making use of these TMS measurements to indicate neurophysiological deficits in MDD patients.

## Data Availability Statement

The raw data supporting the conclusions of this article will be made available by the authors, without undue reservation.

## Ethics Statement

The studies involving human participants were reviewed and approved by the Medical Ethics Review Committee, Erasmus Medical Center, Rotterdam, The Netherlands. The patients/participants provided their written informed consent to participate in this study.

## Author Contributions

JC collected and analyzed the data and wrote the manuscript. JT, TB, SK, and YE contributed to the study design and participated in the interpretation of data. TB contributed to the data collection of the study. All authors revised, read, and approved the submitted version.

## Funding

This work was supported by resources of the Department of Clinical Genetics, Department of Neuroscience, and the Department of Psychiatry of the Erasmus MC, Rotterdam, Netherlands.

## Conflict of Interest

The authors declare that the research was conducted in the absence of any commercial or financial relationships that could be construed as a potential conflict of interest.

## Publisher's Note

All claims expressed in this article are solely those of the authors and do not necessarily represent those of their affiliated organizations, or those of the publisher, the editors and the reviewers. Any product that may be evaluated in this article, or claim that may be made by its manufacturer, is not guaranteed or endorsed by the publisher.
